# Prediction of redox-sensitive cysteines using sequential distance and other sequence-based features

**DOI:** 10.1186/s12859-016-1185-4

**Published:** 2016-08-24

**Authors:** Ming-an Sun, Qing Zhang, Yejun Wang, Wei Ge, Dianjing Guo

**Affiliations:** 1 State Key Laboratory of Agrobiotechnology and School of Life Sciences, The Chinese University of Hong Kong, Shatin, New Territories, Hong Kong, People’s Republic of China; 2 Department of Cell Biology and Genetics, School of Basic Medical Sciences, Shenzhen University Health Science Center, Nanhai Ave 3688, Shenzhen, 518060 People’s Republic of China; 3 Centre of Reproduction, Development and Aging, Faculty of Health Sciences, University of Macau, Taipa, Macau People’s Republic of China

**Keywords:** Reactive oxygen species, Redox-sensitive cysteine, Post-translational modification, Support vector machine, SVM-based recursive feature elimination

## Abstract

**Background:**

Reactive oxygen species can modify the structure and function of proteins and may also act as important signaling molecules in various cellular processes. Cysteine thiol groups of proteins are particularly susceptible to oxidation. Meanwhile, their reversible oxidation is of critical roles for redox regulation and signaling. Recently, several computational tools have been developed for predicting redox-sensitive cysteines; however, those methods either only focus on catalytic redox-sensitive cysteines in thiol oxidoreductases, or heavily depend on protein structural data, thus cannot be widely used.

**Results:**

In this study, we analyzed various sequence-based features potentially related to cysteine redox-sensitivity, and identified three types of features for efficient computational prediction of redox-sensitive cysteines. These features are: sequential distance to the nearby cysteines, PSSM profile and predicted secondary structure of flanking residues. After further feature selection using SVM-RFE, we developed Redox-Sensitive Cysteine Predictor (RSCP), a SVM based classifier for redox-sensitive cysteine prediction using primary sequence only. Using 10-fold cross-validation on RSC758 dataset, the accuracy, sensitivity, specificity, MCC and AUC were estimated as 0.679, 0.602, 0.756, 0.362 and 0.727, respectively. When evaluated using 10-fold cross-validation with BALOSCTdb dataset which has structure information, the model achieved performance comparable to current structure-based method. Further validation using an independent dataset indicates it is robust and of relatively better accuracy for predicting redox-sensitive cysteines from non-enzyme proteins.

**Conclusions:**

In this study, we developed a sequence-based classifier for predicting redox-sensitive cysteines. The major advantage of this method is that it does not rely on protein structure data, which ensures more extensive application compared to other current implementations. Accurate prediction of redox-sensitive cysteines not only enhances our understanding about the redox sensitivity of cysteine, it may also complement the proteomics approach and facilitate further experimental investigation of important redox-sensitive cysteines.

**Electronic supplementary material:**

The online version of this article (doi:10.1186/s12859-016-1185-4) contains supplementary material, which is available to authorized users.

## Background

Reactive oxygen species (ROS) are toxic oxygen-derived molecules generated during various cellular processes [1]. Accumulation of ROS may result in the damage of different cellular components including proteins, nucleic acids, lipids and metal cofactors. It has been indicated that many diseases, including type II diabetes, cancer, neurodegenerative diseases and cardiovascular disease, are associated with oxidative stress [2]. Thus, ROS has traditionally been regarded as unwanted by-products of aerobic metabolism [1]. However, under normal conditions, ROS can modify the structure and function of proteins in defined ways [3–5]. ROS may also act as important signaling molecules in gene transcription and translation, stress protection, apoptosis, metabolism and other processes [6–9]. Reactive nitrogen species (RNS), a family of antimicrobial molecules derived from nitrite oxide (·NO) and superoxide (O_2_^·−^) produced via the enzymatic activity of inducible nitric oxide synthase 2 and NADPH oxidase respectively, also play similar roles as ROS does [10, 11].

Cysteine is of the least abundance among 20 common amino acids. However, cysteine residues usually are more conserved and tend to play critical roles [12, 13]. Cysteine residues bear a thiol group that represents the most reduced state of sulfur in proteins. These thiol groups can be oxidized to disulfide (S-S), sulfenic acid (S-OH), sulfinic acid (SO_2_H), sulfonic acid (SO_3_H), S-nitrosothiol (S-NO) or S-glutathione (S-SG). Sulfenic acids are usually the intermediate of thiol-modification, which can react with other thiols or be further oxidized. Sulfinic acid and sulfonic acid represent the irreversibly oxidized products. Alternatively, sulfenic acids can be oxidized to disulfide bonds or S-nitrosothiols, which can be reduced back to thiols by thioredoxin, glutaredoxin, or glutathione [14, 15]. Redox-sensitive cysteines undergo reversible thiol modifications in response to ROS or RNS, thereby modulate protein function, activity or localization, and serve as a regulatory switch for proteins in response to cellular redox state [2, 15–17].

Traditionally, redox-sensitive cysteines are identified by biochemical characterization of proteins accompanied by site-directed mutagenesis experiment [18, 19]. Over the past decade, tremendous progress in the field of redox proteomics has been made and different gel electrophoresis (DIGE) and isotope coded affinity tag (ICAT) strategies have been used to measure cysteine oxidation [20, 21]. However, proteomics techniques are insensitive to proteins with low abundance including most transcription factors. For this reason, many proteins identified by proteomics techniques to date are components of redox homeostasis systems or highly abundant target proteins such as ribosomal proteins or enzymes [22]. Moreover, proteomics approach is costly. Computational approaches which are not limited by protein abundance can therefore provide an important alternative to proteomics based approaches.

Cysteine could be functionally categorized as structural disulfide bonded Cys, metal-Cys, catalytic Cys and regulatory Cys, with some cysteines belong to multiple groups [23]. Most identified redox-sensitive cysteines are known to function as catalytic or regulatory Cys. Despite the fact that various in *silico* methods have been developed for the prediction of structural disulfide bonded Cys [24–29] and metal-Cys [30–32], computational approaches for predicting redox-sensitive cysteines are limited. Thiol oxidoreductases, which usually bear a dicysteine active site motif (CXXC), are the most extensively studied proteins with catalytic redox-active cysteines. Based on the observation that Sec (selenocysteine) usually locate at the active sites of redox proteins, Fomenko et al. developed a procedure for high-throughput identification of catalytic redox-active Cys by searching for Sec/Cys pairs in sequence databases [33]. In another study, Marino et al. analyzed general features of catalytic redox-active cysteines in thiol oxidoreductases at the structural level, and designed a structure-based method for predicting thiol oxidoreductases and their catalytic redox-active cysteine residues [34]. These approaches are efficient for detecting catalytic redox-active cysteines in thiol oxidoreductase. However, they cannot be used for detecting redox-active cysteines in other protein types.

Apart from catalytic redox-active cysteines, some regulatory cysteines may affect protein activity when oxidized or reduced. Such regulatory redox-sensitive cysteines have been found in transcription factors, kinases, phosphatases, chaperone and other proteins [23, 35]. Compared to catalytic redox-sensitive cysteines, regulatory cysteines are much more difficult to predict. Computational tool that can accurately predict cysteines with regulatory roles is of great importance for our understanding of cysteine thiol oxidation [23]. Sanchez et al. studied various protein structure features and found three features useful for the prediction of redox-sensitive cysteines: distance to the nearest cysteine sulfur atom, solvent accessibility and pKa [36]. Using these features, a decision-tree based classifier Cysteine Oxidation Prediction Algorithm (COPA) was developed for predicting redox-susceptible cysteines [36]. This study provided valuable information about the determinants of cysteine redox-sensitivity. However, the application of COPA is highly limited due to its dependence on protein structure data, which are not available for most proteins in the proteomes. In another study, Fan et al. scanned the Protein Data Bank for potential redox-active cysteine pairs by looking for proteins with alternate redox states [37] and recovered 1,134 unique redox pairs of proteins, many of which exhibit conformational differences between alternate redox states. Again, the structural data for both oxidized and reduced form of protein are required for this method [37]. Such simple and straightforward procedure is useful for scanning the entire Protein Data Bank database; however, it can hardly be used for *de novo* prediction. Computational methods independent of protein structure data is therefore in great need for a better understanding of redox-sensitive cysteines.

In this study, a dataset of experimentally validated redox-sensitive cysteines (RSC758) was collected and various features possibly related to cysteine redox-sensitivity were critically analyzed. Among them, three types of features that are efficient for redox-sensitive cysteine prediction were identified. After further feature selection using SVM-RFE, a corresponding SVM classifier namely RSCP was developed. Using 10-fold cross-validation on RSC758, the model achieved accuracy of 0.679, sensitivity of 0.602, specificity of 0.756, MCC of 0.362 and AUC of 0.727. When evaluated using 10-fold cross-validation with BALOSCTdb dataset which has structure information, the model’s performance was comparable to current structure-based method. The robustness of RSCP was further validated using an independent dataset.

## Results

### Performance using different combinations of features on RSC758 dataset

**Fig. 1:**
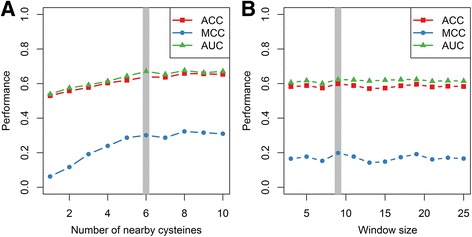
Optimization of the parameters for feature extraction. **a** Performance with different numbers of nearby cysteines. The numbers of nearby cysteines are optimized between 1 and 10. **b** Performance with different window sizes. The window sizes are optimized between 3 and 25. The gray bars indicated the finally selected parameters

Using the RSC758 dataset, we first optimized the parameters for feature extraction, including: 1) the number of nearby cysteines for which the sequential distance is considered; 2) the window size for Position-Specific Scoring Matrix profile (PSSM), predicted secondary structure (SS), predicted solvent accessibility (SA) and physical-chemical property (PCP). The 1) and 2) parameters were optimized separately. We first extracted the sequential distances to the 1^st^ to 10^th^ nearest cysteines, and then SVM classifiers were trained. The performance for different classifiers was compared according to the ACC, MCC and AUC values from using 10-fold cross-validation (Fig. 1a). The best result was achieved when sequential distance for the 1^st^ to 6^th^ nearby cysteines were considered. Similarly, features including PSSM, SS, SA and PCP were extracted using sliding windows with window sizes between 3 and 25, and the performances from 10-fold cross-validation were compared (Fig. 1b). The best performance was achieved when using window size of 9. Thus, sequential distance to the 6th nearby cysteines, and PSSM, SS, SA and PCP features extracted with a window size of 9 were used in the following study.

**Fig. 2:**
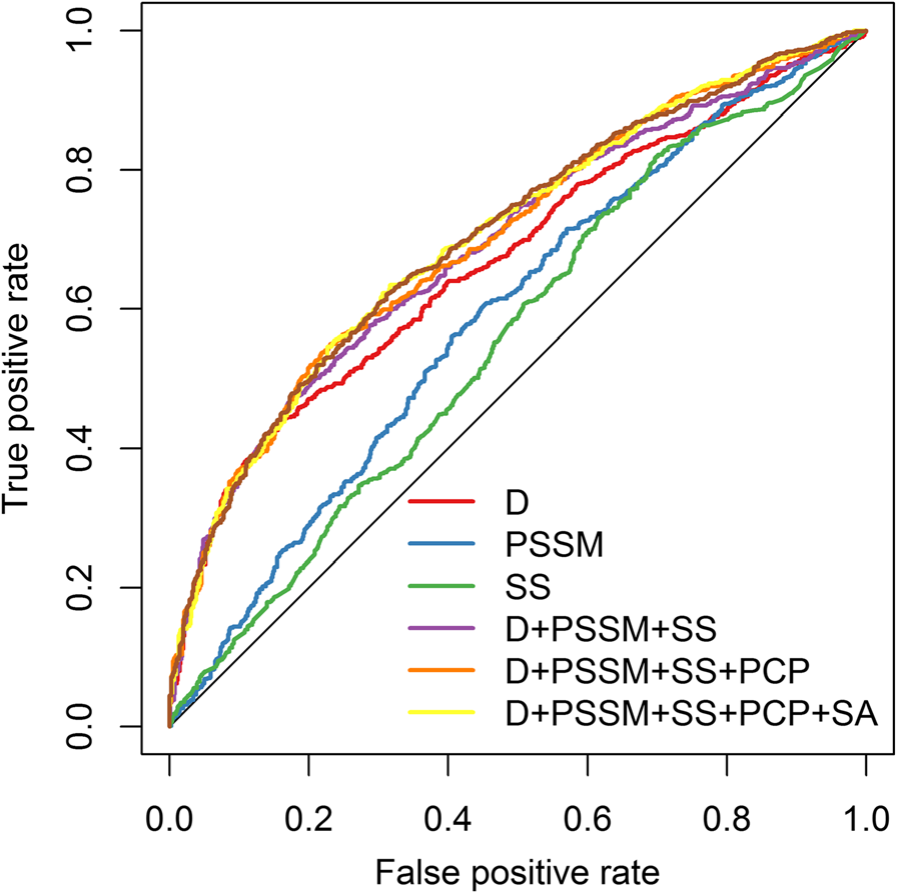
The ROC curves of SVM classifiers using RSC758 dataset. The average values of true positive rate and false positive rate from 10-fold cross-validation are used

The performance using different combinations of features was further compared (Table 1, Fig. 2). When each single type of feature was tested, sequential distance to nearby cysteines (D) and PSSM were found to be the most efficient features. Specifically, when the model was trained using sequential distance features only, an AUC value of 0.671 was achieved. An AUC value of 0.700 was achieved when using D + PSSM + SS feature set. Further integration of SA and PCP features only slightly improved the AUC, but not the ACC (Table 1). Thus, the SA and PCP feature sets were excluded from further analysis. By a grid search using 10-fold cross-validation, the regularization parameter C and the kernel parameter *γ* for SVM classifier were optimized as 0.5 and 0.0078125, respectively. The model trained using the full D + PSSM + SS feature set could achieve the performance with ACC of 0.658, SN of 0.516, SP of 0.801, MCC of 0.330 and AUC of 0.700.

**Table 1:** 10-fold cross-validation of different combinations of features on RSC758

Feature	ACC	SN	SP	MCC	AUC
D + PSSM + SS + SA + PC	0.650	0.540	0.761	0.309	0.705
D + PSSM + SS + PC	0.653	0.529	0.777	0.316	0.705
**D + PSSM + SS**	**0.658**	**0.516**	**0.801**	**0.330**	**0.700**
D + PSSM	0.644	0.503	0.785	0.300	0.691
D	0.639	0.442	0.835	0.301	0.671
SS	0.555	0.770	0.339	0.121	0.559
PSSM	0.575	0.578	0.573	0.150	0.590
SA	0.557	0.552	0.562	0.114	0.554
PCP	0.525	0.611	0.439	0.051	0.542

The results are sorted by AUC value. The feature set in bold was selected as the optimal

*D* sequential distance to adjacent cysteines, *PSSM* PSSM profile, *SS* predicted secondary structure, *SA* predicted solvent accessibility, *PCP* physical-chemical property

### Feature selection using SVM-RFE on RSC758 dataset

**Fig. 3:**
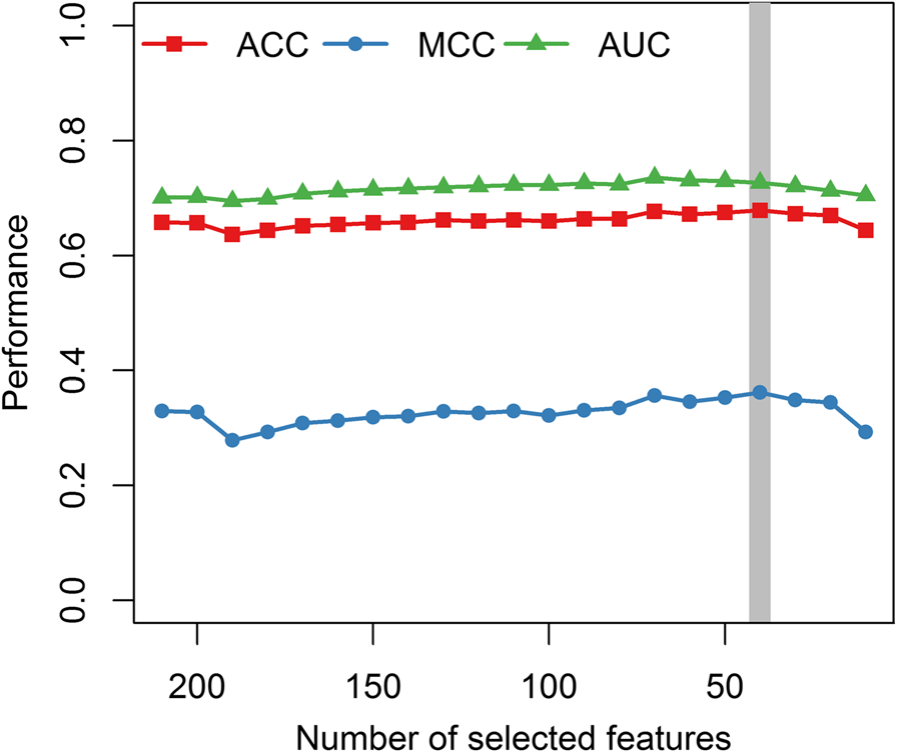
Performance using different number of features selected by SVM-RFE for RSC758 dataset. The x-axis indicated the number of selected features. y-axis represents the ACC, MCC and AUC estimated from 10-fold cross-validation

To further improve the performance, we applied SVM-based Recursive Feature Elimination (SVM-RFE) to the D + PSSM + SS feature set. Initially, the full D + PSSM + SS feature set has 213-dimentional vector. As evaluated by ACC, MCC and AUC estimated from 10-fold cross-validation on RSC758 dataset, the best performance was achieved when utilizing the forty top-ranked features (Fig. 3). By a grid search using 10-fold cross-validation, the regularization parameter C and the kernel parameter *γ* for SVM classifier were optimized as 8.0 and 0.0078125, respectively. The corresponding model achieved ACC of 0.679, SN of 0.602, SP of 0.756, MCC of 0.362 and AUC of 0.727, respectively. Further inspection showed that three features for D, 25 for PSSM and 11 for SS, are among these selected features (Additional file 1: Table S1).

### Performance of different machine learning techniques on RSC758 dataset

**Fig. 4:**
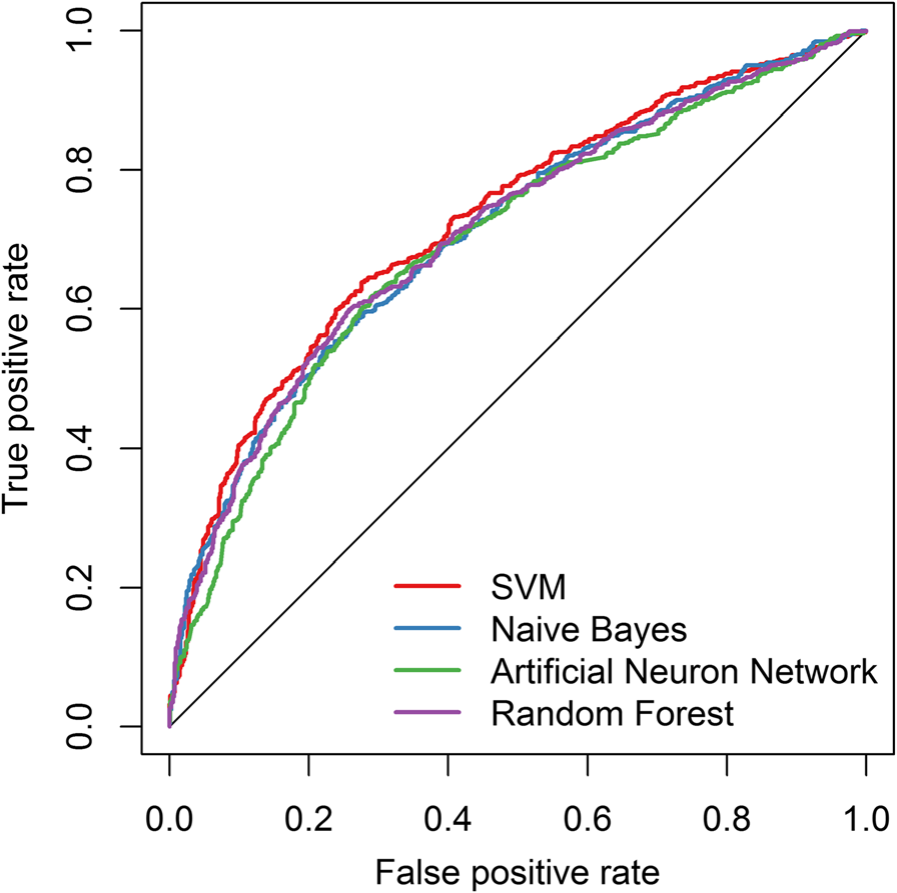
The ROC curves of different machine learning techniques using the forty selected features for RSC758 dataset. The average values of true positive rate and false positive rate from 10-fold cross validation are used

In addition to SVM, we also compared the performance of three other widely used machine learning techniques, including Naive Bayes, Artificial Neural Network and Random Forest. Similarly, the major parameters for these models were tuned by grid searching. Then, the performance was evaluated by 10-fold cross-validation using the forty selected features on RSC758 dataset. The result showed that SVM outperforms other approaches regarding ACC, MCC and AUC (Table 2; Fig. 4). Random Forest was the second best one, with ACC of 0.664, SN of 0.611, SP of 0.718, MCC of 0.330 and AUC of 0.711. Thus, the SVM classifier trained using the forty selected features was used as the final model.

**Table 2:** 10-fold cross-validation with forty selected features using different machine learning methods on RSC758

	ACC	SN	SP	MCC	AUC
SVM	0.679	0.602	0.756	0.362	0.727
Naive Bayes	0.648	0.450	0.846	0.322	0.713
Random Forest	0.664	0.611	0.718	0.330	0.711
Artificial Neural Network	0.662	0.615	0.708	0.325	0.698

The results are sorted by AUC value

### Evaluation of the most efficient features

Our study revealed that three features, including sequential distance to nearby cysteines, PSSM profile and predicted secondary structure, are efficient for redox-sensitive cysteine prediction. We next investigated if redox-sensitive cysteines show distinct patterns when considering these features. Identification of such patterns is of particular importance for our understanding of the determinants of cysteine redox-sensitivity.

**Fig. 5:**
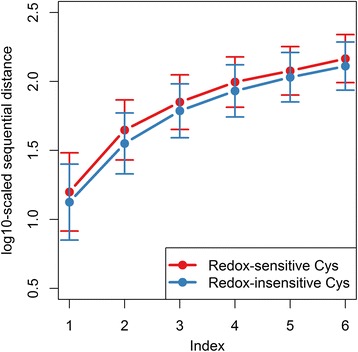
Comparison of sequential distance to nearby cysteines between redox-sensitive and redox-insensitive cysteines. This result is derived from the RSC758 dataset. The x-axis indicated the index of nearby cysteines (for example, 1 indicated the nearest cysteine, and 2 indicates the 2nd nearest cysteine). y-axis represents the log10-scaled sequential distance. The error bars represent the standard deviation

Sequential distance to the nearby cysteines (D) has been previously used to predict structural disulfide [27, 29], another cysteine oxidative state different from reversible oxidation. In this study, we this feature to be the most efficient for predicting redox-sensitive cysteines, indicating that it may be associated with cysteine redox-sensitivity. We found that the sequential distance to nearby cysteines seems to be longer for redox-sensitive cysteines compared with redox-insensitive ones (Fig. 5). From the OSCTdb dataset which has quite different gene family composition, we observed similar pattern except for the sequential distance to the most nearest cysteine (Additional file 2: Figure S1). This is probably due to the fact that more than one third of the proteins (36 from 100) in OSCTdb are thiol oxidoreductases, which usually bear two redox-sensitive cysteines within the typical CXXC motif.

PSSM profile represents the probability of occurrence for each type of amino acid residues, thus it can be considered as a measure of residue conservation in a given location. Using the whole RSC758 dataset, we examined the average PSSM scores of the flanking region for redox-sensitive and redox-insensitive cysteines. We found that several types of amino acids (H, I, L, M, F, P and Y) showed significantly different PSSM scores surrounding redox-sensitive and redox-insensitive cysteines (Paired Student’s *t*-test, Bonferroni corrected *p*-value < 0.05) (Additional file 2: Figure S2). The predicted secondary structure for residues in the flanking region of redox-sensitive and -insensitive cysteines was summarized in Additional file 2: Figure S3. The result indicated an over-represented coil and relatively depleted helix surrounding redox-sensitive cysteines compared with redox-insensitive ones.

### Comparison with structure-based method

The performance of RSCP was compared with Cysteine Oxidation Prediction Algorithm (COPA), which is a decision-tree based classifier using protein structural features [36]. Because RSC758 dataset does not contain structural information, the comparison was conducted using BALOSCTdb dataset by 10-fold cross-validation. WEKA package [38] was used for decision-tree implement, and M value was set to 50 as suggested in the original paper of COPA [36].

The sequential distance, PSSM and SS features were extracted using the same parameters as aforementioned. The regularization parameter C and the kernel parameter *γ* for SVM classifier were optimized as 2.0 and 0.03125, respectively. We first evaluated the performance on BALOSCTdb dataset using the forty features selected according to RSC758 dataset. It achieved an ACC of 0.683, SN of 0.671, SP of 0.696, MCC of 0.362 and AUC of 0.727, which is quite similar to that evaluated on RSC758 dataset (Table 3; Additional file 2: Figure S4A). Because the protein family composition in BALOSCTdb is quite different from RSC758, we further examined the performance using features selected based on BALOSCTdb dataset itself (Additional file 2: Figure S5). The result showed that with the twenty top-ranked features by SVM-RFE, it could achieve the best performance with ACC of 0.761, SN of 0.770, SP of 0.752, MCC of 0.522 and AUC of 0.821 (Table 3; Additional file 2: Figure S4B). Among these twenty features, three are for D, four for PSSM and thirteen for SS (Additional file 1: Table S2).

**Table 3:** Performance comparison between RSCP and COPA using BALOSCTdb by 10-fold cross-validation

	Features	ACC	SN	SP	MCC	AUC
RSCP	40 features selected using RSC758	0.683	0.671	0.696	0.362	0.727
	20 features selected using BALOSCTdb	0.761	0.770	0.752	0.522	0.821
COPA	3 structure based features	0.786	0.776	0.795	0.572	0.823

In summary, the result showed that even without structure data, the performance of RSCP could still be comparable to COPA (Table 3, Additional file 2: Figure S4). This also indicated that although cysteine redox-sensitivity is mainly affected by protein structural environment as revealed by some previous studies [39–41], redox-sensitive cysteines can still be inferred using only sequence features with moderate accuracy. Sequence-based prediction is much more advantageous in that it is not limited by structure data thus can be widely used for prediction as long as the protein sequence data is available.

### Performance evaluation using OSCTdb

We further evaluated the performance of RSCP trained from RSC758 using OSCTdb as independent dataset. OSCTdb consists of a few types of enzymes together with some non-enzyme proteins, and catalytic redox-active cysteines from oxidoreductase make up about one third of the dataset. However, when collecting RSC758, we tried not to be biased towards any gene families by including various types of enzymes and a large number of non-enzyme proteins. RSCP achieved ACC of 0.629, SN of 0.789, SP of 0.561 and MCC of 0.322 (Table 4), using this independent dataset. Even though the testing dataset has very different protein family composition to that of training dataset, the prediction accuracy on this independent testing dataset is similar to the cross-validation result, indicating RSCP is robust.

**Table 4:** Performance evaluation using OSCTdb by gene families

Protein class	#Cys	ACC	SN	SP	MCC
Oxidoreductase	175	0.606	0.815	0.482	0.297
Hydrolase	110	0.736	0.783	0.724	0.424
Transferase	96	0.479	0.739	0.397	0.121
Non-enzyme proteins	124	0.718	0.784	0.690	0.435
Total	537	0.629	0.789	0.561	0.322

Only gene families with at least ten redox-sensitive cysteines were shown

The testing dataset also contain different gene family composition compared to the training dataset. The prediction accuracy on different gene families was summarized in Table 4. The result indicates that RSCP is robust on predicting redox-sensitive cysteines in most gene families except for transferases. RSCP achieved the highest accuracy of 0.736 for hydrolases. Redox-sensitive cysteines have been identified mainly from enzymes, especially oxidoreductases. A variety of non-enzyme proteins are also regulated via redox processes. Although regulatory cysteines are thought to be much difficult to predict [23], RSCP achieved high accuracy of 0.718 for cysteines in non-enzyme proteins. This model is therefore particularly useful for the analysis of regulatory redox-sensitive cysteines.

## Discussion

Thiol-based redox regulation and signaling has become one of the important research focuses in recent years. In this study, we identified three important sequence-based features that are efficient for the prediction of redox-sensitive cysteines. After feature selection using SVM-RFE, we further developed a sequence-based SVM classifier for predicting redox-sensitive cysteines. When evaluated with BALOSCTdb dataset which has structure information, the model achieved performance comparable to current structure-based method. The major advantage of this sequence-based classifier lays in its independence of protein structure data, which is not readily available for a large portion of the proteomes.

The high reactivity and chemical plasticity of cysteine, mainly due to its sulfur-based functional group, has been well known [42]. For redox-sensitive cysteine, those could form reversible disulfides, are most well studied [43–45]. Unlike structural disulfides which cannot be easily opened once formed, reversible disulfides could be reversibly oxidized and reduced under different conditions thus function as regulatory switches. In this study, cysteines forming reversible disulfides were considered as redox-sensitive ones, while those forming structural disulfides were included in the the negative training dataset. By compiling the training dataset in this way, we expected the trained model could also have potential ability to distinguish these two types of disulfide-bonded cysteines.

Apart from redox sensitivity as we focused in this study, cysteines could also function via binding different metal irons [46] such as Fe^2+/3+^ and Zn^2+^. A number of previous studies as review in [47, 48] suggested that some well known zinc-factor binding cysteines could also undergo redox modification. When generating the training dataset, redox-sensitive cysteines with metal-binding function were not excluded. We neither tried to distinguish redox-sensitive cysteines with or without metal-binding potential for analysis. However, in the future, it would be interesting to investigate how the determinants of redox-sensitivity and metal-binding potential for cysteine are related.

Two datasets were used in this study: BALOSCTdb is a dataset adopted from previous studies which is smaller but with structural information, and RSC758 is a newly generated dataset of larger size and relatively unbiased. While the model optimized and evaluated using BALOSCTdb could achieve good performance comparable to current structure-based method, the model trained using RSC758 dataset only achieved moderate accuracy. One possibility is that apart from the redox-sensitivity, cysteines under different types of redox modifications also have their distinct properties which are not well represented by the identified features. In this study, we aimed at examine the common features underlying redox-sensitivity, and develop a general purpose predictor of redox-sensitive cysteines. But with the accumulation of validated redox-sensitive cysteines, it would be interesting to perform comparative analysis among different types of redox modification to reveal their unique features. It is also highly desirable to develop computational tools which could not only predict the redox sensitivity but also the exact type of redox modification.

## Conclusions

In this study, we identified three important sequence-based features for redox-sensitive cysteines, and further developed a SVM classifier for predicting redox-sensitive cysteines. We expect the accurate prediction of redox-sensitive cysteines could not only enhance our understanding about the redox sensitivity of cysteine, but also complement the proteomics approach and facilitate further experimental investigation of important redox-sensitive cysteines.

## Methods

### Datasets

The RSC758 dataset (Additional file 1: Table S3), which contains proteins with redox-sensitive cysteines, was obtained by searching literatures and public databases [49, 50]. When generating the dataset, we tried not to bias towards oxidoreductase, which is the most well studied gene family in terms of redox-sensitive cysteines. All the sequences were retrieved from SWISSPROT/UNIPROT [50], and these sequences are mainly from mammals, bacteria, plant, algae, yeast and some parasites. Various types of reversible thiol modification including reversible disulfide, sulfenic acid, S-nitrosothiol and S-glutathione are included in this dataset. BLASTClust [51] was used to remove sequences that share more than 25 % similarity with each other. The non-redundant dataset contains 456 protein sequences with 758 redox-sensitive cysteines. Remaining cysteines that are not reported as redox-sensitive in these proteins are regarded as redox-insensitive. Each cysteine was then labelled as 1 (redox-sensitive) or −1 (redox-insensitive). We randomly chose 758 redox-insensitive cysteines to form a balanced dataset. Notably, all the sequences in RSC758 dataset are of less than 25 % similarity to OSCTdb sequences.

Oxidation Susceptible Cysteine Thiol Database (OSCTdb) comprises 100 proteins with 161 redox-sensitive cysteines [36]. All the sequences are of less than 35 % identity to each other. Equal numbers of redox-insensitive cysteines were included as negative data to form a balanced OSCTdb (BALOSCTdb). BALOSCTdb was used to compare the model performance. When used as independent dataset to evaluate the performance of RSCP, we retrieved all the cysteines from these sequences to form a testing dataset. This dataset includes all the cysteines occurred in those proteins and therefore represents the real situation for prediction.

### Feature extraction

#### PSSM profiles

The PSSM profiles were generated using PSI-BLAST [51] against the NCBI non-redundant (NR) database by three iterations of search under default settings. The PSI-BLAST as implemented in the blastpgp execute was used to generated PSSM profiles with parameter “-j 3 ”. We then extracted the feature with a local sliding window to produce a feature vector represented as a matrix of *L × 20* with a window size of *L*. For visualization, we averaged between the PSSM profiles for each cysteines along with the flanking regions, then visualized the averaged matrix as heatmap.

#### Sequential distance to nearby cysteines

The sequential distance to nearby cysteines has been previous used for predicting disulfide bonded cysteines [27, 29]. The sequential distance between two cysteines is defined as:

1 $$ D\left(i,j\right)=\left|i-j\right| $$

where *i* and *j* represent the position of two cysteines in a protein sequence. For each cysteine, the sequential distance to its *n*th nearest cysteines was defined as *D*_*n*_. Here we used the absolute values without further normalization.

#### Predicted secondary structure and solvent accessibility

We used SSpro [52, 53] to predict the secondary structure (SS) and solvent accessibility (SA) of each residue. Three secondary structure states (helix, strand and coil) were denoted as “H”, “E” and “C”, respectively. The predicted secondary structure is extracted using a local sliding window, and represented as a *L × 3* vector with a window size of *L*. Similarly, exposed and buried residues were denoted as “E” and “B”, then represented as a *L × 2* vector. The frequency of different types of predicted secondary structure surrounding redox-sensitive and redox-insensitive cysteines were illustrated using WebLogo [54].

#### Physical-chemical property

We extracted four types of amino acid physical-chemical property (PCP) including hydrophobicity [55], net charge index of side chains of amino acids (NCI) [56], propensity and side chain pKa value. These features have been successfully used for predicting RNA-binding sites in proteins [57]. Those features were extracted using local sliding window, and were represented as a *L × 4* vector with window size of *L*. The physical-chemical property for each amino acid residue can be found in Additional file 1: Table S4.

### Support vector machines (SVMs) implementation and parameter optimization

Support vector machine (SVM) [58] is a widely used machine-learning method based on statistical learning theory. In this work, SVM technique was implemented using LIBSVM 3.20 [59]. The radial basis function (RBF kernel) is used, which is defined as:

2 $$ K\left({x}_i,x\right)= \exp \left(-\gamma \left\Vert {x}_i-x\right\Vert \right) $$

where *x* and *x*_*i*_ are two data vectors and *γ* is a training parameter. The regularization parameter C and the kernel parameter *γ* were optimized by a grid search approach using 10-fold cross-validation.

### SVM-RFE

SVM Recursive Feature Elimination (SVM-RFE) [60] has been widely used to rank features and to select the significant ones for classification. In a sequentially backward elimination manner, SVM-RFE ranks the features by the change in objective function when removing one feature. The ranking iteration will be terminated when all features are ranked. Notably, with eliminating a feature in each step of the SVM-RFE procedure, the error rate caused by the eliminating feature was determined by an independent testing dataset in contrast to the training dataset. In this study, we adopted the SVM-RFE procedure as implement in the “Feature selection with SVM-RFE” MATLAB scripts for feature selection [61].

### Implementation and parameter optimization for other machine learning techniques

In this study, we also examined the performance of three other widely used machine learning techniques, including Naive Bayes [62], Artificial Neural Network [63] and Random Forest [64]. Naive Bayes algorithm was implemented by the e1071 (version 1.6-7) R package [62], with the Laplace smoothing be optimized. The Artificial Neural Network was implemented in the Nnet R package [63], with number of units in the hidden layer (size) and parameter for weight decay (decay) tuned using the wrapper in e1071 R package. The random forest algorithm was implemented by the randomForest R package [65], with the main parameters, including minimum size of terminal nodes (nodesize) and number of trees grown (ntree), tuned using the wrapper in e1071 R package.

### Performance assessment

The performance is evaluated using different criteria including sensitivity (SN), specificity (SP), accuracy (ACC) and Matthews correlation coefficient (MCC). They are defined as below:

3 $$ SN=\frac{TP}{TP+FN} $$

4 $$ SP=\frac{TN}{TN+FP} $$

5 $$ ACC=\frac{TP+TN}{TP+TN+FP+FN} $$

6 $$ MCC=\frac{\left(TP\times TN\right)-\left(FP\times FN\right)}{\sqrt{\left(TP+FP\right)\times \left(TP+FN\right)\times \left(TN+FP\right)\times \left(TN+FN\right)}} $$

where TP, TN, FP, and FN denotes the numbers of true positives, true negatives, false positives, and false negatives, respectively.

The model’s performance was evaluated using 10-fold cross-validation. The receiver operating characteristic (ROC) curve, which is one of the most robust approaches for classifier evaluation, was obtained by plotting true positive rate (sensitivity) against the false positive rate (1-specificity). The area under the ROC curve (AUC) was also calculated.

### Web server implementation

The web server is implemented using Perl, PHP and Apache. With the optimized parameters using RSC758 dataset by 10-fold cross-validation, a SVM classifier based on the forty features selected by SVM-RFE was trained for the web server. The web server and all the data used in this study are freely available at: http://biocomputer.bio.cuhk.edu.hk/RSCP.

## Additional files

Additional file 1:
**Table S1.** Ranks of top forty features selected by SVM-RFE using RSC758 dataset. **Table S2.** Ranks of top twenty features selected by SVM-RFE using BALOSCTdb dataset. **Table S3.** RSC758 dataset used in this study. **Table S4.** Physical-chemical properties for each amino acid residue. (XLSX 81 kb) Additional file 2:
**Figure S1.** Comparison of sequential distance to nearby cysteines between redox-sensitive and redox-insensitive cysteines using BALOSCTdb dataset. The *x*-axis indicated the index of nearby cysteines (for example, 1 indicated the nearest cysteine, and 2 indicates the 2^nd^ nearest cysteine). *y*-axis represents the log10-scaled sequential distance. **Figure S2.** PSSM profile of residues flanking redox-sensitive cysteines and redox-insensitive cysteines. This result was derived from the RSC758 dataset. A and B illustrate the PSSM profile for the flanking region of redox-sensitive and redox-insensitive cysteines, respectively. The PSSM profiles are calculated from the RSC758 dataset, and this figure is drawn according to the average value. Amino acid types are labelled at the bottom, and the relative position to the corresponding cysteine residues are labelled on the right. **Figure S3.** Predicted secondary structure of surrounding residues. The frequency of different types of predicted secondary structure surrounding redox-sensitive cysteines (A) and redox-insensitive cysteines (B) are shown. *x*-axis indicates the relative residue position to cysteine; *y*-axis indicates the frequency of predicted secondary structure. **Figure S4.** The ROC curve of SVM classifier based on 10-fold cross-validation using BALOSCTdb dataset. A. Top forty features selected by SVM-RFE on RSC758 dataset were used. B. Top twenty features selected by SVM-RFE on BALOSCTdb itself were used. **Figure S5.** Performance using different number of features selected by SVM-RFE for BALOSctdb dataset. The x-axis indicated the number of selected features. y-axis represents the ACC, MCC and AUC estimated from 10-fold cross-validation. (PDF 605 kb)
