# Supplementary material for: Prediction of redox-sensitive cysteines using sequential distance and other sequence-based features
Source: BMC Bioinformatics. 2016 Aug 24;17(1):316. doi: 10.1186/s12859-016-1185-4 (PMC4995733; doi:10.1186/s12859-016-1185-4)
Supplement: Additional file 2: — Figure S1. Comparison of sequential distance to nearby cysteines between redox-sensitive and redox-insensitive cysteines using BALOSCTdb dataset. The x-axis indicated the index of nearby cysteines (for example, 1 indicated the nearest cysteine, and 2 indicates the 2nd nearest cysteine). y-axis represents the log10-scaled sequential distance. Figure S2. PSSM profile of residues flanking redox-sensitive cysteines and redox-insensitive cysteines. This result was derived from the RSC758 dataset. A and B illustrate the PSSM profile for the flanking region of redox-sensitive and redox-insensitive cysteines, respectively. The PSSM profiles are calculated from the RSC758 dataset, and this figure is drawn according to the average value. Amino acid types are labelled at the bottom, and the relative position to the corresponding cysteine residues are labelled on the right. Figure S3. Predicted secondary structure of surrounding residues. The frequency of different types of predicted secondary structure surrounding redox-sensitive cysteines (A) and redox-insensitive cysteines (B) are shown. x-axis indicates the relative residue position to cysteine; y-axis indicates the frequency of predicted secondary structure. Figure S4. The ROC curve of SVM classifier based on 10-fold cross-validation using BALOSCTdb dataset. A. Top forty features selected by SVM-RFE on RSC758 dataset were used. B. Top twenty features selected by SVM-RFE on BALOSCTdb itself were used. Figure S5. Performance using different number of features selected by SVM-RFE for BALOSctdb dataset. The x-axis indicated the number of selected features. y-axis represents the ACC, MCC and AUC estimated from 10-fold cross-validation. (PDF 605 kb) [file 12859_2016_1185_MOESM2_ESM.pdf]

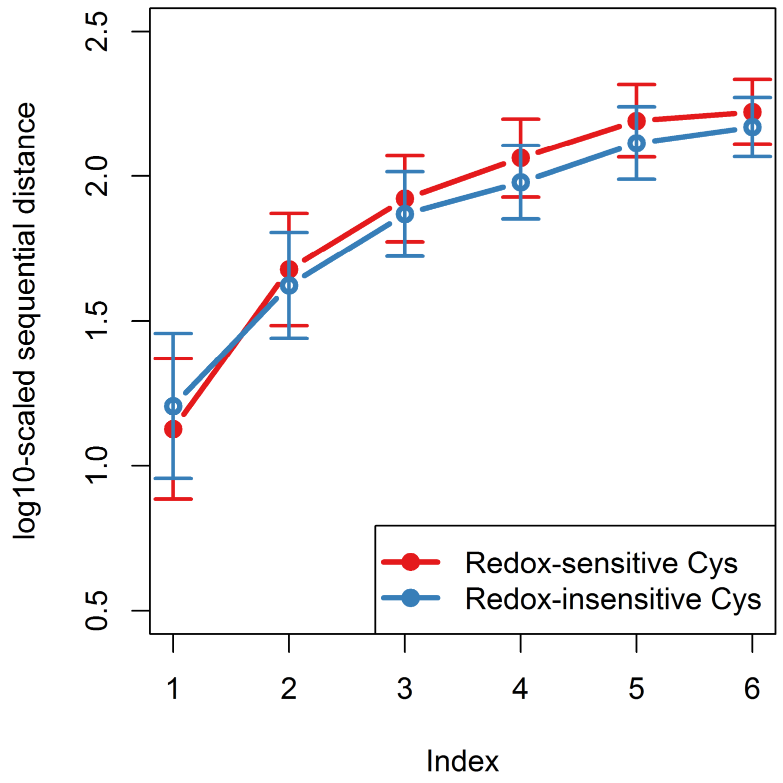

Figure S1 Comparison of sequential distance to nearby cysteines between redox-sensitive and redox-insensitive cysteines using BALOSCTdb dataset. The *x*-axis indicated the index of nearby cysteines (for example, 1 indicated the nearest cysteine, and 2 indicates the 2nd nearest cysteine). *y*-axis represents the log10-scaled sequential distance.

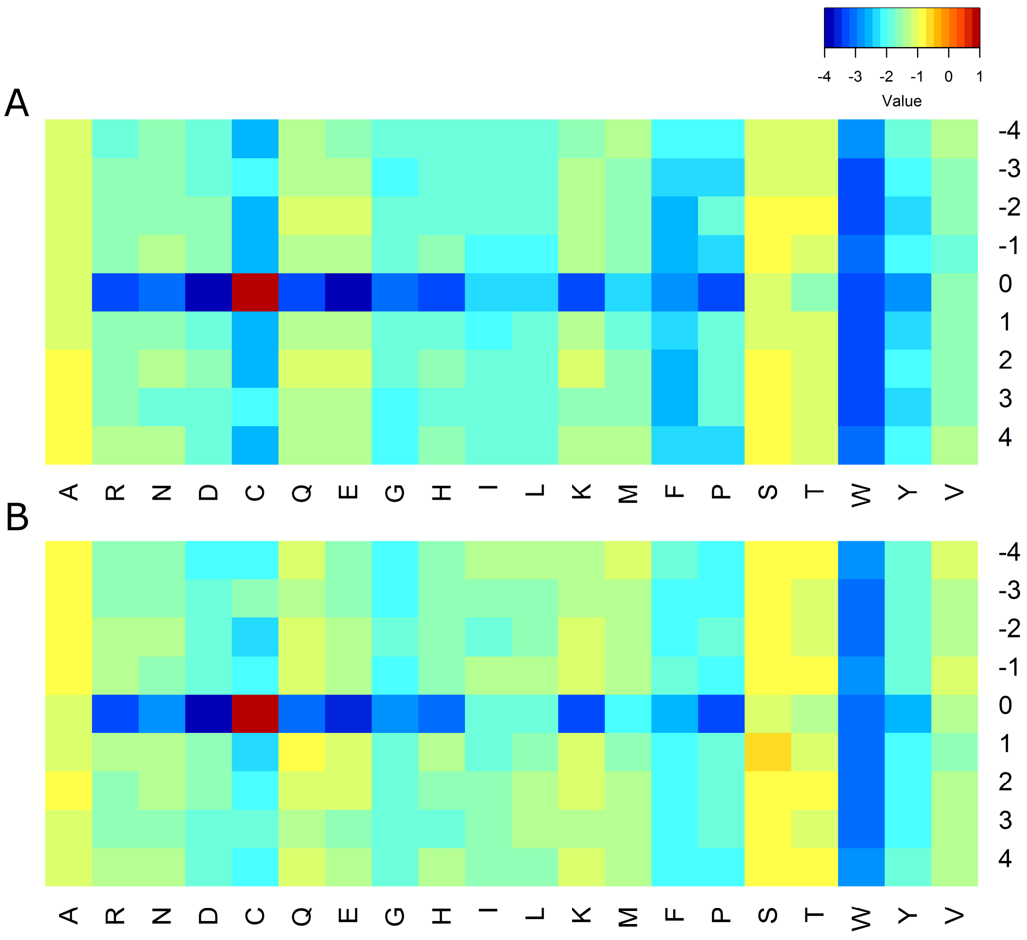

Figure S2 PSSM profile of residues flanking redox-sensitive cysteines and redox-insensitive cysteines. This result was derived from the RSC758 dataset. A and B illustrate the PSSM profile for the flanking region of redox-sensitive and redox-insensitive cysteines, respectively. The PSSM profiles are calculated from the RSC758 dataset, and this figure is drawn according to the average value. Amino acid types are labelled at the bottom, and the relative position to the corresponding cysteine residues are labelled on the right.

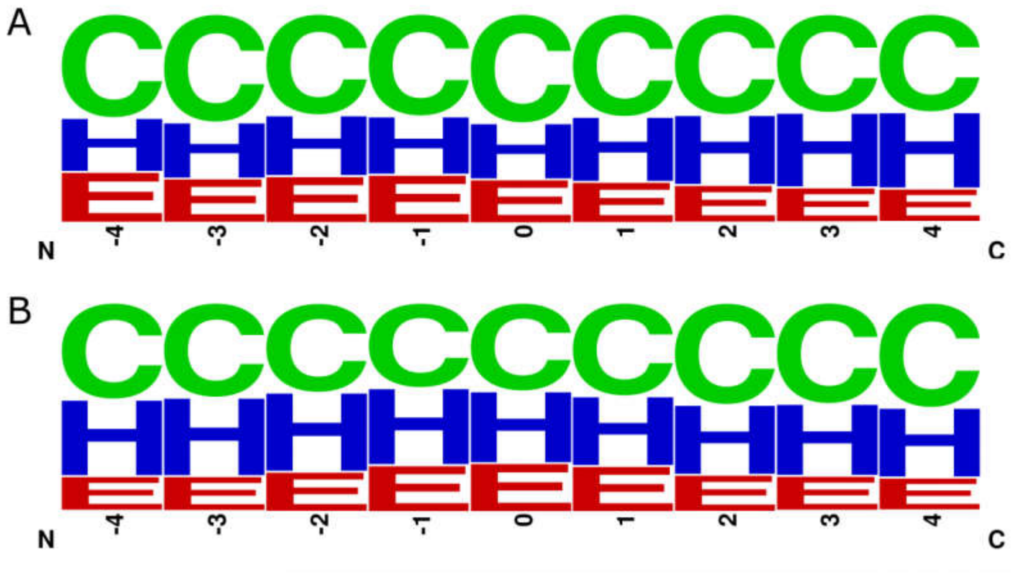

Figure S3 Predicted secondary structure of surrounding residues. The frequency of different types of predicted secondary structure surrounding redox-sensitive cysteines (A) and redox-insensitive cysteines (B) are shown. *x*-axis indicates the relative residue position to cysteine; *y*-axis indicates the frequency of predicted secondary structure.

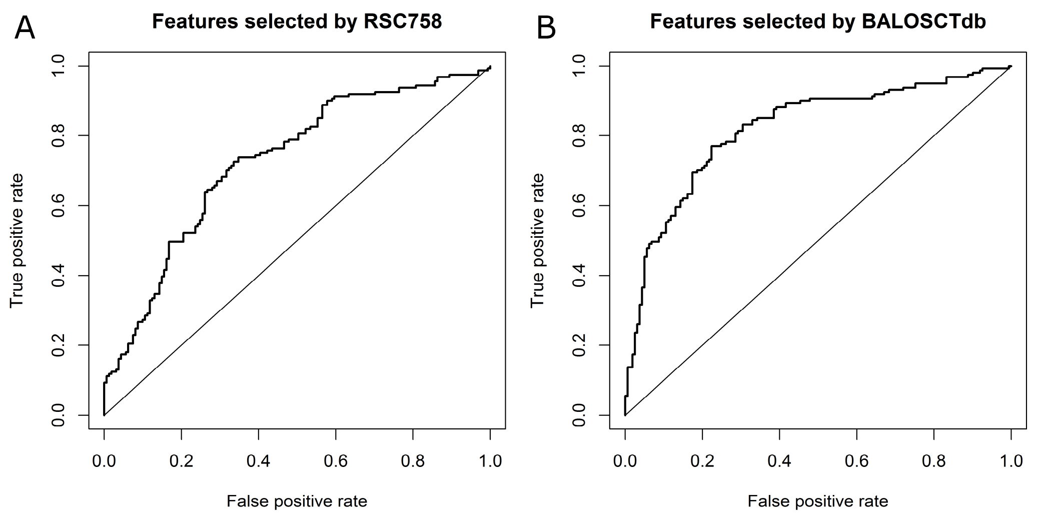

Figure S4 The ROC curve of SVM classifier based on 10-fold cross-validation using BALOSCTdb dataset. A. Top forty features selected by SVM-RFE on RSC758 dataset were used. B. Top twenty features selected by SVM-RFE on BALOSCTdb itself were used.

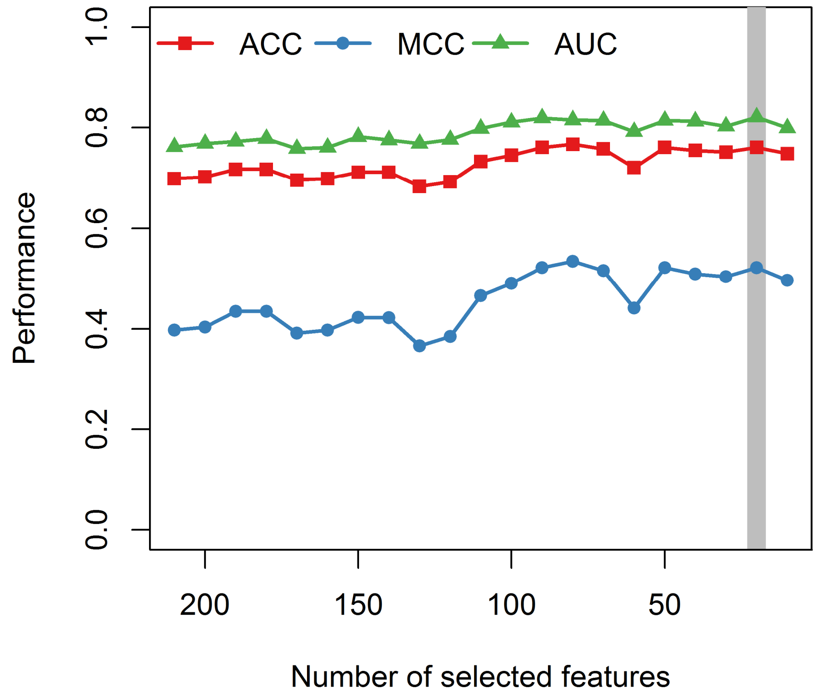

Figure S5 Performance using different number of features selected by SVM-RFE for BALOSctdb dataset. The x-axis indicated the number of selected features. y-axis represents the ACC, MCC and AUC estimated from 10-fold cross-validation.
